# Bayesian Uncertainty Quantification with Multi-Fidelity Data and Gaussian Processes for Impedance Cardiography of Aortic Dissection

**DOI:** 10.3390/e22010058

**Published:** 2019-12-31

**Authors:** Sascha Ranftl, Gian Marco Melito, Vahid Badeli, Alice Reinbacher-Köstinger, Katrin Ellermann, Wolfgang von der Linden

**Affiliations:** 1 Institute of Theoretical Physics-Computational Physics, Graz University of Technology, 8010 Graz, Austria; 2 Institute of Mechanics, Graz University of Technology, 8010 Graz, Austria; gmelito@tugraz.at (G.M.M.); ellermann@tugraz.at (K.E.); 3 Institute of Fundamentals and Theory in Electrical Engineering, Graz University of Technology, 8010 Graz, Austria; vahid.badeli@tugraz.at (V.B.); alice.koestinger@tugraz.at (A.R.-K.)

**Keywords:** uncertainty quantification, multi fidelity, Gaussian processes, probability theory, Bayes, impedance cardiography, aortic dissection

## Abstract

In 2000, Kennedy and O’Hagan proposed a model for uncertainty quantification that combines data of several levels of sophistication, fidelity, quality, or accuracy, e.g., a coarse and a fine mesh in finite-element simulations. They assumed each level to be describable by a Gaussian process, and used low-fidelity simulations to improve inference on costly high-fidelity simulations. Departing from there, we move away from the common non-Bayesian practice of optimization and marginalize the parameters instead. Thus, we avoid the awkward logical dilemma of having to choose parameters and of neglecting that choice’s uncertainty. We propagate the parameter uncertainties by averaging the predictions and the prediction uncertainties over all the possible parameters. This is done analytically for all but the nonlinear or inseparable kernel function parameters. What is left is a low-dimensional and feasible numerical integral depending on the choice of kernels, thus allowing for a fully Bayesian treatment. By quantifying the uncertainties of the parameters themselves too, we show that “learning” or optimising those parameters has little meaning when data is little and, thus, justify all our mathematical efforts. The recent hype about machine learning has long spilled over to computational engineering but fails to acknowledge that machine learning is a big data problem and that, in computational engineering, we usually face a little data problem. We devise the fully Bayesian uncertainty quantification method in a notation following the tradition of E.T. Jaynes and find that generalization to an arbitrary number of levels of fidelity and parallelisation becomes rather easy. We scrutinize the method with mock data and demonstrate its advantages in its natural application where high-fidelity data is little but low-fidelity data is not. We then apply the method to quantify the uncertainties in finite element simulations of impedance cardiography of aortic dissection. Aortic dissection is a cardiovascular disease that frequently requires immediate surgical treatment and, thus, a fast diagnosis before. While traditional medical imaging techniques such as computed tomography, magnetic resonance tomography, or echocardiography certainly do the job, Impedance cardiography too is a clinical standard tool and promises to allow earlier diagnoses as well as to detect patients that otherwise go under the radar for too long.

## 1. Introduction

While Uncertainty Quantification (UQ) has become a term on its own in the computational engineering community, Bayesian Probability Theory is not widely spread yet. A comprehensive collection of reviews on the various methods and aspects of UQ from the point of view of the computational engineering and applied mathematics community can be found in Reference [1]. In References [2,3,4], a statistician’s perspective is discussed. The computational effort for performing UQ with brute force is typically prohibitively large; thus, surrogate models such as Polynomial Chaos Expansion (PCE) [5,6,7,8] or Gaussian Process (GP) regression [9,10,11,12] are used, the latter of which has had its renaissance recently from within the machine learning community.

This work is inspired by the article of Kennedy and O’Hagan in 2000 [13]. They performed UQ by making use of a computer simulation with different levels of “fidelity”, “sophistication”, “accuracy”, or “quality”. In other words, a cheap, simplified simulation serves as a surrogate. We will refer to this approach as Multi-Fidelity scheme (MuFi). The idea of MuFi [14], and MuFi with GPs specifically [15], recently found increasing attention again. In contrast to previously reported MuFi-GPs , we do not learn the parameters and subsequently neglect the parameter uncertainties but explicitly incorporate them in a rigorous manner. We find that this is tractable analytically for all parameters but especially for the ones that occur nonlinearly or inseparably in the GPs covariance.

While UQ in general has arrived fully in the biomedical engineering community [16], the Bayesian approach has not. Biehler et al. [17] were, to the best knowledge of the authors, the first to apply a Bayesian MuFi Scheme in the context of computational biomechanical UQ. We apply our method to quantify the uncertainties in finite element simulations [18] of Impedance Cardiography (ICG) [19] of Aortic Dissection (AD) [20]. The aorta is the largest blood vessel in the human body. In aortic dissection, blood fluid dynamics force open a tear in a weakened aortic wall, dilate it, and fill the wall itself with blood. This deforms the geometry of the aorta and, obviously, affects blood circulation unfavourably (p. 459, [21]). Aortic Dissection is highly dangerous and likely lethal if untreated. Thus, a fast response and, hence, a fast diagnosis are key to the treatment of patients. For diagnosis, physicians use a variety of imaging techniques such as Magnetic Resonance Tomography (MRT), Computed Tomography (CT), and Echocardiography [22]. Echocardiography performed by a trained cardiologist is comparably cheap and fast, yet sound wave propagation might be hindered, e.g., by the rib cage or body fat. In CT and MRT, the radiation fully penetrates the body. Still, they require a trained radiologist and long measurement times and pose radiation risks and high costs. Most importantly, these examinations are not performed without a specific reason.

Alternatively, impedance cardiography is rather cheap and simple and, more importantly, available in any clinic and many medical practices. In ICG, one places a pair of electrodes on the thorax (upper body), injects a defined low-amplitude, alternates electric current into the body, and measures the voltage drop. The specific resistance of blood is much lower than that of muscle, fat, or bone [23]. Since electric current seeks the path of least resistance, the current propagates through the aorta rather than through, e.g., the spine. Thus, if the local blood volume changes due to aortic dissection, the impedance signal changes as well. Impedance cardiography could therefore complement existing clinical procedures and could detect aortic dissection when medical imaging is not performed, be it due to the absence of suspicion or to the unavailablity of the device itself. We find a number of parameters which are well defined but usually neither known precisely nor accessible in the clinical setting, e.g., the size of the aortic dissection. A clinical trial is extremely difficult, and we resort to a theoretical investigation instead, in which we account for the uncertainties as well.

In Section 2, we develop a Bayesian uncertainty quantification model based on Gaussian processes using multi-fidelity data. We scrutinize the method with mock data in Section 3 and show that learning regression parameters has little meaning when data is little. In Section 4, we apply our method to finite element simulations of impedance cardiography of aortic dissection and show that low-fidelity data can indeed decrease high-fidelity uncertainties.

## 2. Bayesian Multi-Fidelity Scheme

### 2.1. Statistical Model

Let C be the conditional complex. Let $t=1,...,N_{t}$ denote the ranked levels of fidelity of a simulation, with level $N_{t}$ being the highest fidelity. $z_{t}\left(x_{t}\right)$ is a vector of simulation results of fidelity-level *t* given input vector $x_{t}$. We assume that $z_{t}\left(x_{t}\right)$ is a realisation of a Gaussian Process (GP) $z_{t}\left(x_{}\right)$ with a Markov property of order 1, meaning that level *t* depends on level t−1 only via the following recursive relationship:

(1a) $$\begin{array}{cc} z_{1}\left(x_{}\right) & =\delta_{1}\left(x_{}\right) \end{array}$$

(1b) $$\begin{array}{cc} z_{t}\left(x_{}\right) & =\rho_{t-1}z_{t-1}\left(x_{}\right)+\delta_{t}\left(x_{}\right)\;\;\forall t\geq 2 \end{array}$$

(1c) $$\begin{array}{cc} \Rightarrow z_{N_{t}}\left(x_{}\right) & =\sum_{t=1}^{N_{t}}\delta_{t}\left(x_{}\right)\prod_{l=t}^{N_{t}-1}\rho_{l} \end{array}$$

with a “difference-GP” $\delta_{t}\left(x_{}\right)$ and a proportionality constant $\rho_{t-1}$. Further, we assume that all information about a level is contained in the data corresponding to the same pivot point at that level and its previous level. Formally, that is $Cov\left(z_{t}\left(x_{}\right),z_{t-1}\left(x^{\prime }\right)|z_{t-1}\left(x_{}\right)\right)=0$. The difference-GP $\delta_{t}\left(x_{}\right)$ shall be defined by the covariance matrix $\sigma_{t}^{2}K_{t}\left(x,x^{\prime }\right)$ and the mean function $h_{t}\left(x\right)\beta_{t}$. $h_{t}\left(x\right)$ is a matrix of regression functions $h_{t}^{\left(k\right)}$ evaluated at $x={\left(x^{\left(1\right)},...,x^{\left(j\right)},...,x^{\left(N_{x}\right)}\right)}^{T}$ with size $N_{x}\times N_{\beta_{t}}$, where $N_{x}$ is the length of input vector $x_{}$ and $N_{\beta_{t}}$ is the expansion power, i.e., number of regression functions, at level *t*. $\beta_{t}={\left(\beta_{t}^{\left(1\right)},...,\beta_{t}^{\left(N_{\beta_{t}}\right)}\right)}^{T}$ are the coefficients of level *t*’s regression functions, and $\alpha_{t}$ is the set of hyperparameters parametrizing the kernel function $k_{t}$. Formally, this is

(2a) $$\begin{array}{cc} p\left[\delta_{t}\left(x_{}\right)|C\right] & =N(h_{t}\left(x\right)\beta_{t},\sigma_{t}^{2}K_{t}\left(x,x\right)) \end{array}$$

(2b) $$\begin{array}{cc} {\left[h_{t}\left(x\right)\right]}_{jk} & =h_{t}^{\left(k\right)}\left(x^{\left(j\right)}\right) \end{array}$$

(2c) $$\begin{array}{cc} {\left[h_{t}\left(x\right)\beta_{t}\right]}_{j} & =\sum_{k=1}^{m}h_{t}^{\left(k\right)}\left(x^{\left(j\right)}\right)\beta_{t}^{\left(k\right)} \end{array}$$

(2d) $$\begin{array}{cc} {\left[K_{t}\left(x,x\right)\right]}_{ij} & =k_{t}\left(x^{\left(i\right)},x^{\left(j\right)};\alpha_{t}\right) \end{array}$$

At this point, neither have we chosen the basis functions $h_{t}^{\left(k\right)}$ building the mean function nor have we chosen the kernel functions $k_{t}$ building the covariance matrices. Let us subsume the parameters as $\theta =\{\theta_{t}\}$, $\theta_{t}=\left\{\beta_{t},\sigma_{t},\rho_{t-1},\alpha_{t}\right\}$, $\beta_{}=\left\{\beta_{t}\right\},\beta_{t}=\left\{\beta_{t}^{\left(k\right)}\right\}$, $\sigma_{}=\left\{\sigma_{t}\right\}$, $\rho_{}=\left\{\rho_{t}\right\}$, and $\alpha_{}=\left\{\alpha_{t}\right\}$ with $t=1,2,...,N_{t}$. The data shall be $D=\{D_{t}\}$ with $D_{t}=\left\{\left(x_{t},z_{t}\left(x_{t}\right),z_{t-1}\left(x_{t}\right)\right)\right\}$, which comprises the input vector at level *t*, namely $x_{t}$, and its corresponding computer code outputs at level *t*, namely $z_{t}\left(x_{t}\right)$, and at the previous level t−1, namely $z_{t-1}\left(x_{t}\right)$. Further, we require a nested design of input vectors, i.e., $x_{N_{t}}\subseteq x_{N_{t}-1}\subseteq ...x_{t}\subseteq x_{t-1}...\subseteq x_{1}$. We want to draw conclusions from the predictive posterior probability of $z_{N_{t}}\left(x_{}\right)$ at a set of points *x*,

$$\begin{array}{cc} p\left[z_{N_{t}}\left(x_{}\right)|D\right] & =\int \prod_{t=1}^{N_{t}}d\delta_{t}\left(x_{}\right)\int d\theta \;p\left[z_{N_{t}}\left(x_{}\right)|{\left\{\delta_{t}\left(x_{}\right)\right\}}_{t=1}^{N_{t}},\theta ,D\right]p\left[{\left\{\delta_{t}\left(x_{}\right)\right\}}_{t=1}^{N_{t}}|\theta ,D\right]p\left[\theta |D\right] \end{array}$$

This thing is quite unhandy. We will instead just deal with its moments only, namely the posterior mean $\langle z_{N_{t}}\left(x_{}\right)\rangle$ and the posterior covariance $Cov\left(z_{N_{t}}\left(x_{}\right)\right)=\langle z_{N_{t}}\left(x_{}\right)z_{N_{t}}{\left(x_{}\right)}^{T}\rangle -\langle z_{N_{t}}\left(x_{}\right)\rangle {\langle z_{N_{t}}\left(x_{}\right)\rangle }^{T}$, where the diagonal of the posterior covariance is the uncertainty band of the prediction. The moments of $z_{N_{t}}\left(x_{}\right)$ follow from

(3) $$\begin{array}{cc} \langle f(z_{N_{t}}\left(x_{}\right))\rangle & =\int dz_{N_{t}}\left(x_{}\right)f\left(z_{N_{t}}\left(x_{}\right)\right)p\left[z_{N_{t}}\left(x_{}\right)|D\right]\\ \; & =\int \prod_{t=1}^{N_{t}}d\delta_{t}\left(x_{}\right)\int \prod_{t=1}^{N_{t}}d\theta_{t}f\left(\sum_{t=1}^{N_{t}}\delta_{t}\left(x_{}\right)\prod_{l=t}^{N_{t}-1}\rho_{l}\right)\prod_{t=1}^{N_{t}}p\left[\delta_{t}\left(x_{}\right)|\theta_{t},D_{t}\right]p\left[\theta_{t}|D_{t}\right] \end{array}$$

We have used the fact that $p\left[z_{N_{t}}\left(x_{}\right)|{\left\{\delta_{t}\left(x_{}\right)\right\}}_{t=1}^{N_{t}},\theta ,D\right]$ reduces to Dirac’s delta-distribution since $z_{N_{t}}\left(x_{}\right)$ is uniquely determined by Equation (1) and the knowledge of all difference-GPs, ${\left\{\delta_{t}\left(x_{}\right)\right\}}_{t=1}^{N_{t}}$. Thus, integration with respect to $z_{N_{t}}\left(x_{}\right)$ is merely a replacement of $z_{N_{t}}\left(x_{}\right)\to \sum_{t=1}^{N_{t}}\delta_{t}\left(x_{}\right)\prod_{l=t}^{N_{t}-1}\rho_{l}$. Per construction, we can factor $p\left[{\left\{\delta_{t}\left(x_{}\right)\right\}}_{t=1}^{N_{t}}|\theta ,D\right]p\left[\theta |D\right]=\prod_{t}p\left[\delta_{t}\left(x_{}\right)|\theta_{t},D_{t}\right]p\left[\theta_{t}|D_{t}\right]$. Since $\delta_{t}\left(x_{}\right)$ is assumed to obey a GP, the prior probability of $\delta_{t}\left(x_{t}\right)$ is multivariate normal. If the likelihood $p\left[D_{t}|\delta_{t}\left(x_{}\right),\theta_{t}\right]$ is Gaussian, then the posterior probability of $\delta_{t}\left(x_{t}\right)$, $p\left[\delta_{t}\left(x_{t}\right)|\theta_{t},D_{t}\right]$, is multivariate normal as well. Integration with respect to $\delta_{t}\left(x_{}\right)$ yields thus the standard result of the posterior mean value (see, e.g., Reference [10]) and results in a replacement of the GP with its posterior mean.

### 2.2. Prediction and Its Uncertainty

To compute posterior mean and covariance, we are thus left with integration with respect to the hyperparameters. This is done analytically with all parameters ($\beta_{},\sigma_{},\rho_{}$) but the parameters of the kernel function, $\alpha_{}$, since those usually occur nonlinearly in the kernel function. We are then left with numerical integration of expectations and covariances, both conditioned on $\alpha_{}$. We assume flat priors for $\beta_{}$ and $\rho_{}$ and Jeffreys’ prior for $\sigma_{}$, i.e., $p\left[\sigma_{t}|C\right]=\frac{1}{\sigma_{t}}$. The prior of $\alpha_{}$ needs to be chosen only after the covariance kernel has been chosen. Let $\langle \cdot |\alpha_{t}\rangle$ denote the expectation value conditioned on $\alpha_{}$. Here, for ease of notation, we will instead write $\langle \cdot \rangle$ only. The technicalities shall be detailed in the Appendix A , and the result is as follows:

(4a) $$\begin{array}{cc} \langle z_{N_{t}}\left(x_{}\right)\rangle & =\int \sum_{t=1}^{N_{t}}\langle \delta_{t}\left(x_{}\right)\rangle \prod_{l=t}^{N_{t}-1}\langle \rho_{l}\rangle p\left[\alpha_{}|D\right]d\alpha_{}\\ \langle z_{N_{t}}\left(x_{}\right){\left(z_{N_{t}}\left(x_{}\right)\right)}^{T}\rangle & =\int \sum_{t=1}^{N_{t}}\left(\langle \sigma_{t}^{2}\rangle \Sigma_{t}\left(x,x\right)+\langle \delta_{t}\left(x_{}\right)\rangle {\langle \delta_{t}\left(x_{}\right)\rangle }^{T}\right)\prod_{l=t}^{N_{t}-1}\langle \rho_{l}^{2}\rangle p\left[\alpha_{}|D\right]d\alpha_{}\\ \langle \delta_{t}\left(x_{}\right)\rangle & =h_{t}\left(x\right)\langle \beta_{t}\rangle +K_{t}\left(x_{},x_{t}\right)K_{t}{\left(x_{t},x_{t}\right)}^{-1}\left(\delta_{t}\left(x_{t}\right)-h_{t}\left(x_{t}\right)\langle \beta_{t}\rangle \right)\\ \Sigma_{t} & =\left(K_{t}\left(x_{},x_{}\right)-K_{t}\left(x_{},x_{t}\right)K_{t}{\left(x_{t},x_{t}\right)}^{-1}K_{t}\left(x_{t},x_{}\right)\right)\\ p\left[\alpha_{}|D\right] & =\frac{2^{-N_{t}}}{\sqrt{\Phi_{1}}}\frac{\Gamma (\gamma_{1})}{\Gamma (\gamma_{1}-\frac{1}{2})}\prod_{t=1}^{N_{t}}a_{t}^{-\frac{1}{2}}{\left(\pi \Phi_{t}\right)}^{-\gamma_{t}+\frac{1}{2}}\Gamma \left(\gamma_{t}-\frac{1}{2}\right){\left[\frac{\left|K_{t}\left(x_{t},x_{t}\right)\right|}{\left|A_{t}\right|}\right]}^{-\frac{1}{2}}p\left[\alpha_{}|C\right] \end{array}$$

where $\delta_{t}\left(x_{t}\right)$ is determined from the data and Equation (2), Γ(·) is the complete Gamma-function, ∣·∣ is the matrix determinant, and the conditional expectations of the hyperparameters are

(4b) $$\begin{array}{cccc} \langle \beta_{t}\rangle & =A_{t}t\left(z_{t}\left(x_{t}\right)-\langle \rho_{t-1}\rangle z_{t-1}\left(x_{t}\right)\right) & \langle \rho_{t-1}\rangle & =\frac{b_{t}}{a_{t}}\\ \langle \sigma_{t}^{2}\rangle & =\frac{\Phi_{t}}{2\gamma_{t}-3+3\delta_{t1}} & \langle \rho_{t-1}^{2}\rangle & =\frac{\langle \sigma_{t}^{2}\rangle }{a_{t}}+{\left(\frac{b_{t}}{a_{t}}\right)}^{2} \end{array}$$

where $\delta_{t1}=1$ for t=1 and 0 otherwise and the abbreviations are

(4c) $$\begin{array}{cccc} \gamma_{t} & =\frac{N_{x_{t}}-N_{\beta_{t}}}{2} & \Phi_{t} & =c_{t}-\frac{b_{t}^{2}}{a_{t}}\\ C_{t} & =K_{t}{\left(x_{t},x_{t}\right)}^{-1}-t^{T}A_{t}t & c_{t} & ={\left(z_{t}\left(x_{t}\right)\right)}^{T}C_{t}z_{t}\left(x_{t}\right)\\ B_{t} & ={h_{t}\left(x_{t}\right)}^{T}K_{t}{\left(x_{t},x_{t}\right)}^{-1} & b_{t} & ={\left(z_{t}\left(x_{t}\right)\right)}^{T}C_{t}z_{t-1}\left(x_{t}\right)\\ A_{t} & ={\left({\left(h_{t}\left(x_{t}\right)\right)}^{T}K_{t}{\left(x_{t},x_{t}\right)}^{-1}\left(h_{t}\left(x_{t}\right)\right)\right)}^{-1} & a_{t} & ={\left(z_{t-1}\left(x_{t}\right)\right)}^{T}C_{t}z_{t-1}\left(x_{t}\right) \end{array}$$

$N_{x_{t}}$ is the number of pivot points in input vector $x_{t}$, and $N_{\beta_{t}}$ is the expansion order of level *t*’s mean function. For t=1, we need to define $\rho_{0}=0$, $a_{1}=1$, and $b_{1}=0$. Quite importantly, we find following the requirement:

(4d) $$\begin{array}{cc} N_{\beta_{1}} & <N_{x_{1}}-2\\ N_{\beta_{t}} & <N_{x_{t}}-3\;\;\;\;\forall t\geq 2 \end{array}$$

since otherwise the second moments of $\sigma_{t}$ are not defined. The numerical evaluation of this result merely involves a couple of matrix operations. The only input is the data, regression functions, and covariance matrices. No parameters need to be tuned.

## 3. Algorithm and Mock Data Scrutiny

We test the method for the special case of two levels. Then, posterior mean and covariance are simply

$$\begin{array}{cc} \langle z_{2}\left(x_{}\right)\rangle & =\int \langle \delta_{1}\left(x_{}\right)\rangle p\left[\alpha_{1}|D_{1}\right]d\alpha_{1}+\int \langle \rho_{1}\rangle \langle \delta_{2}\left(x_{}\right)\rangle p\left[\alpha_{2}|D_{2}\right]d\alpha_{2}\\ \langle z_{2}\left(x_{}\right){\left(z_{2}\left(x_{}\right)\right)}^{T}\rangle & =\int \left(\langle \sigma_{1}^{2}\rangle \Sigma_{1}\left(x,x\right)+\langle \delta_{1}\left(x_{}\right)\rangle {\langle \delta_{1}\left(x_{}\right)\rangle }^{T}\right)p\left[\alpha_{1}|D_{1}\right]d\alpha_{1}\\ \; & +\int \langle \rho_{1}^{2}\rangle \left(\langle \sigma_{2}^{2}\rangle \Sigma_{2}\left(x,x\right)+\langle \delta_{2}\left(x_{}\right)\rangle {\langle \delta_{2}\left(x_{}\right)\rangle }^{T}\right)p\left[\alpha_{2}|D_{2}\right]d\alpha_{2} \end{array}$$

**Figure 1:**
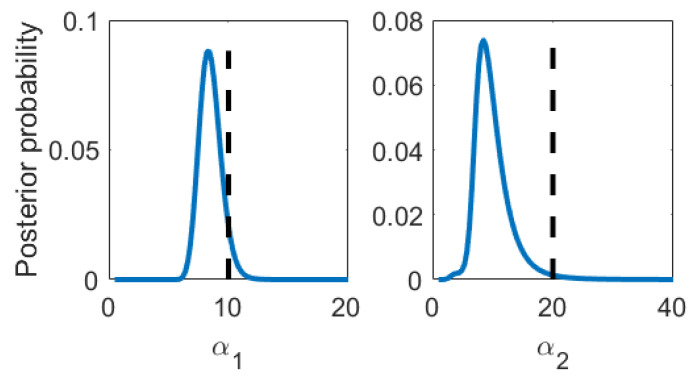
Mock data analysis: Posterior probability density functions of the nonlinear kernel parameters $\alpha_{1}$ and $\alpha_{2}$. Black dashed line: True value

**Figure 2:**
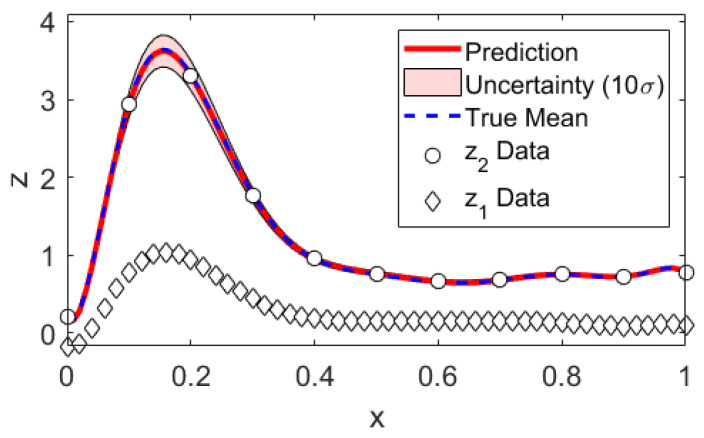
Mock data analysis: Prediction. Note that the uncertainties have been multiplied by a factor of 10 for illustrative purposes.

We generate mock data according to Equations (1) and (2) and compare the data analysis results to the underlying truth. We have chosen as mean function bases the Legendre polynomials up to orders 10 and 4 for Levels 1 and 2, respectively. This is convenient since this basis is both orthogonal as well as normalized on [−1,1] already and the map onto the desired domain is trivial. The covariance kernel was chosen to be the squared exponential kernel, where $\alpha_{1}$ and $\alpha_{2}$ were defined as the inverse of the correlation length squared. This choice was inspired by the typical form of signals encountered in impedance cardiography, about which we will talk more in Section 4. The data set was one sample drawn per level; see Figure 2 . We chose Jeffreys’ prior for $\alpha_{t}$. The integration bounds can be read from Figure 1, and an integration grid of 100 × 100 equally sized volumes turned out to be well converged. As an intermediate result, we compare the multi-fidelity estimates to the true parameter values in Table 1. The posterior probabilities of $\beta_{t},\rho_{t},\sigma_{t}$ are Gaussian. Since $\alpha_{1}$ and $\alpha_{2}$ are not and thus cannot be reasonably well described with mean and variance only, we additionally show their posterior probabilities in Figure 1. The predictions and prediction uncertainties are compared to the true mean in Figure 2. We find that both the parameter estimates as well as the predictions statistically match the truth within their uncertainties. The mean function parameter uncertainties ($\beta_{}$) clearly illustrate that learning parameters by optimization has little meaning if there is little data. As the data set grows big, the posterior will contract to the maximum likelihood solution. Still, and luckily, the prediction uncertainties are kept low because the mean function parameter uncertainties do not appear in the prediction uncertainties directly. We emphasize that our proposed method naturally is applied to little data problems.

Since on level 2 we only have 11 data points, the expansion order of the mean function is limited to a maximum of 7 according to Equation (4d). Unsurprisingly, the solution rapidly worsens as we approach this constraint and entirely breaks down as we reach it because the posteriors become nonconclusive. This is exactly where we find the strength of our multi-fidelity approach. We can choose a high-order mean function on a level where data is abundant and a low-order mean function on a level which we are actually interested in but where data is scarce. The trick is thus actually that the difference of the levels can be modeled by a low-order mean function.

For the sake of completeness, we report the converged log-evidence to be 430±10. In real-life applications of the method, one should and could compare different choices of mean function expansions and covariance kernel functions by Bayesian model comparison [24], i.e., compute each choice’s evidence. Let $N_{\alpha_{t}}$ be the number of hyperparameters in kernel function $k_{t}$. Since $p\left[\alpha_{}|D\right]$ factorizes, the integrals are $N_{\alpha_{t}}$-dimensional each rather than one single integral of dimension $\prod_{t}N_{\alpha_{t}}$, making the computation of the evidence relatively easy. In our case, numerical Riemann integration was good enough. When choosing more sophisticated kernel functions with more hyperparameters, one might need to use statistical integration methods such nested sampling [25], which conveniently and automatically yields the evidence as well.

For the sake of numerical stability, it is advisable to rescale the data. Further, one might want to improve the condition numbers of the prior covariance matrices, $\sigma_{t}^{2}K_{t}\left(x,x\right)$, by adding a small term proportional to the identity matrix, where the proportionality constant should be several orders of magnitude smaller than $\sigma_{t}^{2}$.

Finally, we would like to point out that Equation (4) suggests trivial parallelisation of the code levels. This is not easily recognizable in the presentation of Kennedy and O’Hagan [3] but was found by Le Gratiet and Garnier [26] already.

The algorithm, implemented in Matlab (R2019a), shall be available on https://github.com/Sranf/Bayesian-MuFi-GP.git.

## 4. Application to Finite Element Simulations of Impedance Cardiography of Aortic Dissection

In this section, we quantify the uncertainties of real simulation data. We compare the uncertainties of our Bayesian MuFi-GP with normal Bayesian GP neglecting the additional LoFi data, that is, the special case of $N_{t}=1$ in Equation (4). We have described the physical and physiological model in our previous work [20,27] but shall restate a brief summary here for the reader’s convenience.

**Figure 3:**
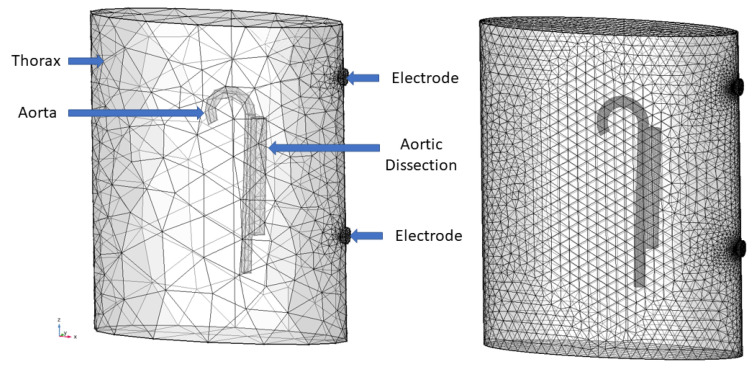
Right: Mesh-converged HiFi model with 100,000–550,000 degrees of freedom. Left: LoFi model with 9000–15,000 with labels of the geometrical objects. Adapted from Reference [27]

We solve Laplace’s equation:

$$\nabla \cdot \left((\sigma +i\omega \varepsilon )\nabla V\right)=0\;,$$

with finite elements on a geometry depicted in Figure 3, where *V* is the electric potential, σ is the electrical conductivity (not to be confused with the regression parameter in the GP kernel), ω is the angular current frequency, ε is the permittivity, and *i* is the imaginary unit. We had von-Neumann boundary conditions, where V=const. on the top electrode and V=0 on the bottom electrode, where the surface integral of the current was held constant at 4 mA and air was assumed to be perfectly insulating. The current had a frequency of 100 kHz. We considered one cardiac cycle that spans one second. The dynamics were modelled via a time-dependent radius of the aorta and its dissection, which arises from pressure waves in a pulsatile flow. Further, the blood conductivity is parametrized in time via its dependence on flow velocity. In the dissected aorta, we assume flow to be stagnant [20,28]. The voltage drop is then measured from just below the top electrode to just above the bottom electrode. The impedance is then the ratio of voltage over current, and the admittance is the inverse of the impedance. We used Comsol Multiphysics for the modelling [29].

For the uncertainty quantification, we chose to expand the mean functions in Legendre polynomials up to order 8 and 2 for HiFi and LoFi, respectively. We choose the squared exponential kernel for both covariance matrices. In principle, one should compute the evidence for a number of plausible choices and choose the one with the most evidence. For the mean function, that would most simply be different expansion orders, while the covariance kernel could be taylored to the PDE at hand to enforce physical behaviour, as suggested in References [30,31].

For uncertain parameters, we consider the radius of the dissected aorta and perform a number of simulations with sensible values within the physiological and physical range, i.e., 1.0–24.0 mm. The LoFi data set consisted of 24 time series, each with 21 pivot points in time. The HiFi data set consisted of 3 time series (5 mm, 11 mm, and 18 mm), each with 11 pivot points in time.

**Figure 4:**
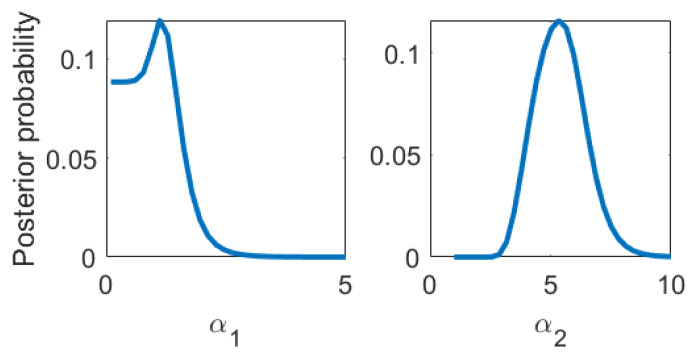
Posterior probability of the nonlinear kernel parameters.

**Figure 5:**
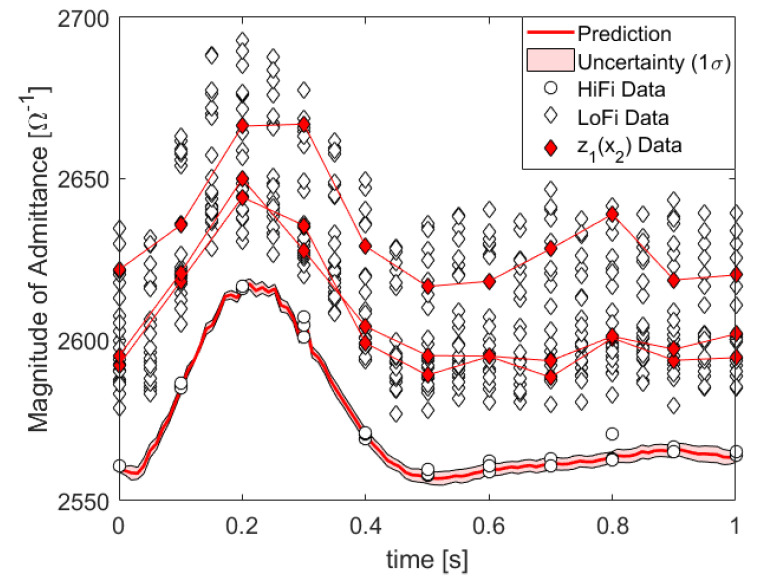
Data, prediction, and prediction uncertainty of the absolute value of the admittance, i.e., the inverse impedance in units of inverse Ohm: $z_{1}\left(x_{2}\right)$ denotes LoFi data at the same pivot points as HiFi data.

In Figure 4, we show the posterior of the kernel parameters, which turns out to be quite conclusive. In Figure 5, we plot the HiFi predictions and uncertainties which are enhanced by LoFi data and compare them to HiFi predictions and uncertainties which are not enhanced by LoFi data as well as to the test data set.

## 5. Conclusions

We devised a fully Bayesian multi-level Gaussian process model to improve uncertainty quantification of expensive and little high-fidelity simulation data by augmenting the data set with low-fidelity simulations. Our proposed method is rigorous and logically consistent, no ad hoc assumptions have been made, and the user is spared the embarrassment of having to tune any parameters. The method was scrutinized with mock data and shown to work with as little data as where simple Bayesian Gaussian process regression is not conclusive at all. We applied the method to finite element simulations of impedance cardiography of aortic dissection and quantified the uncertainty due to the unknown size of the aortic dissection. By using meshes of both high fidelity (defined by mesh convergence) and low fidelity, we reduced the uncertainty significantly. We have thus further shown that uncertainties due to geometrical parameters can be described with Gaussian processes on each level of fidelity. With a coarsened mesh, the result is qualitativelybut not quantitatively similar. Usually, that is not good enough and the low-fidelity data is entirely useless to the engineer. Here we show that this is not necessarily true in the context of uncertainty quantification. Ultimately, we want to diagnose aortic dissection from impedance cardiography signals, i.e., in the parlance of probability theory, we need to compare the evidences of healthy and diseased aortae. Unambiguous judgement will most likely, if at all, be possible only with several electrodes at once.

## Figures and Tables

**Table 1:** Mock data analysis: Comparison of the hyperparameter estimates with their true values.

Hyperparameter	Estimate (Multi-Fidelity)	Truth
$\beta_{1}^{\left(1\right)}$	0.30±0.07	0.32
$\beta_{1}^{\left(2\right)}$	−0.30±0.09	−0.40
$\beta_{1}^{\left(3\right)}$	0.02±0.08	0.1
$\beta_{1}^{\left(4\right)}$	0.34±0.06	0.35
$\beta_{1}^{\left(5\right)}$	−0.50±0.04	−0.51
$\beta_{1}^{\left(6\right)}$	0.35±0.02	0.33
$\beta_{1}^{\left(7\right)}$	−0.033±0.008	−0.034
$\beta_{1}^{\left(8\right)}$	−0.146±0.005	−0.142
$\beta_{1}^{\left(9\right)}$	0.1745±0.0008	0.1750
$\beta_{1}^{\left(10\right)}$	−0.1031±0.0004	−0.1034
$\beta_{2}^{\left(1\right)}$	0.42±0.59	$0.\dot{3}$
$\beta_{2}^{\left(2\right)}$	−0.17±0.55	0.15
$\beta_{2}^{\left(3\right)}$	0.11±0.17	$0.01\dot{6}$
$\beta_{2}^{\left(4\right)}$	0.03±0.10	0
$\sigma_{1}$	0.133±0.096	0.1
$\sigma_{2}$	0.47±0.58	0.01
$\rho_{1}$	2.97±0.02	3
$\alpha_{1}$	8.6±0.9	10.1
$\alpha_{2}$	10±3	20.1

## Sample Availability

The two-level code implemented in Matlab is available on https://github.com/Sranf/Bayesian-MuFi-GP.git. The ICG data is available from the authors upon reasonable request.

## Appendix A. Mathematical Proofs

We start from Equation (3) and want to compute Equation (4) from it. We reintroduce the notation of conditional expectations, where 〈Q∣P〉 is the expectation of *Q* given some *P*, i.e., the integration is done with respect to all parameters but *P*.

$$\begin{array}{cc} p\left[\delta_{t}\left(x_{t}\right)|x_{t},\theta_{t}\right] & =N\left[\delta_{t}\left(x_{t}\right)|{\bar{\delta }}_{t}\left(x_{t}\right),K_{t}\left(x_{t},x_{t}\right)\right]\\ {\bar{\delta }}_{t}\left(x\right) & =h_{t}\left(x\right)\beta_{t}+K_{t}\left(x_{},x_{t}\right)K_{t}{\left(x_{t},x_{t}\right)}^{-1}\left(\delta_{t}\left(x_{t}\right)-h_{t}\left(x_{t}\right)\beta_{t}\right)\\ \Rightarrow p\left[\theta |D\right] & =\prod_{t}p\left[\theta_{t}|D_{t}\right]\\ p\left[D_{t}|\theta_{t}\right] & =N\left[\delta_{t}\left(x_{t}\right)|{\bar{\delta }}_{t}\left(x_{t}\right),K_{t}\left(x_{t},x_{t}\right)\right] \end{array}$$

Thus, in the moments of $z_{N_{t}}\left(x_{}\right)$, by integration with respect to $\delta_{t}\left(x_{}\right)$, we can replace $\delta_{t}\left(x_{}\right)$ by its posterior mean:

$$\begin{array}{cc} \langle z_{N_{t}}\left(x_{}\right)\rangle & =\int d\theta \left(\sum_{t=1}^{N_{t}}{\bar{\delta }}_{t}\left(x\right)\prod_{l=t}^{N_{t}-1}\rho_{l}\right)p\left[\theta |D\right]d\theta \\ \langle z_{N_{t}}\left(x_{}\right)z_{N_{t}}{\left(x_{}\right)}^{T}\rangle & =\int d\theta \left(\sum_{t=1}^{N_{t}}{\bar{\delta }}_{t}\left(x\right)\prod_{l=t}^{N_{t}-1}\rho_{l}\right){\left(\sum_{t=1}^{N_{t}}{\bar{\delta }}_{t}\left(x\right)\prod_{l=t}^{N_{t}-1}\rho_{l}\right)}^{T}p\left[\theta |D\right]d\theta \end{array}$$

### Appendix A.1. Parameter Posterior and Parameter Estimates

We need the posterior of the parameters:

(A1a) $$\begin{array}{cc} N\left[\delta_{t}\left(x_{t}\right)|{\bar{\delta }}_{t}t,K_{t}\left(x_{t},x_{t}\right)\right] & =\frac{1}{Z_{t}}e^{-\frac{\psi_{t}}{2\sigma_{t}^{2}}}\\ \psi_{t} & ={\left(\delta_{t}\left(x_{t}\right)-h_{t}\left(x_{t}\right)\beta_{t}\right)}^{T}K_{t}{\left(x_{t},x_{t}\right)}^{-1}\left(\delta_{t}\left(x_{t}\right)-h_{t}\left(x_{t}\right)\beta_{t}\right)\\ Z_{t} & ={\left(2\pi \sigma_{t}^{2}\right)}^{N_{x_{t}}/2}{\left|K_{t}\left(x_{t},x_{t}\right)\right|}^{1/2} \end{array}$$

The exponent of this Gaussian is a quadratic form in $\beta_{t}$, and we rewrite it as

$$\begin{array}{cc} -\frac{\psi_{t}}{2\sigma_{t}^{2}} & =-\frac{1}{2\sigma_{t}^{2}}{\left(\beta_{t}-\langle \beta_{t}|\alpha_{t},\rho_{t-1}\rangle \right)}^{T}A_{t}^{-1}\left(\beta_{t}-\langle \beta_{t}|\alpha_{t},\rho_{t-1}\rangle \right)-\frac{1}{2\sigma_{t}^{2}}\delta_{t}{\left(x_{t}\right)}^{T}C_{t}\delta_{t}\left(x_{t}\right)\\ \langle \beta_{t}|\alpha_{t},\rho_{t-1}\rangle & =A_{t}B_{t}\left(z_{t}\left(x_{t}\right)-\rho_{t-1}z_{t}\left(x_{t-1}\right)\right)\\ C_{t} & =K_{t}{\left(x_{t},x_{t}\right)}^{-1}-t^{T}A_{t}t\\ B_{t} & ={\left(h_{t}\left(x_{t}\right)\right)}^{T}K_{t}{\left(x_{t},x_{t}\right)}^{-1}\\ A_{t} & ={\left({\left(h_{t}\left(x_{t}\right)\right)}^{T}K_{t}{\left(x_{t},x_{t}\right)}^{-1}\left(h_{t}\left(x_{t}\right)\right)\right)}^{-1} \end{array}$$

From this form, we can read the (conditional) expectation and covariance of $\beta_{t}$. We can now integrate with respect to $\beta_{t}$. We assume a flat prior for $\beta_{t}$:

$$\begin{array}{cc} p\left[\alpha_{t},\sigma_{t},\rho_{t-1}|D_{t}\right] & =\int p\left[\alpha_{t},\sigma_{t},\rho_{t-1},\beta_{t}|D_{t}\right]d\beta_{t}\\ \; & =\exp\left(-\frac{1}{2\sigma_{t}^{2}}\delta_{t}{\left(x_{t}\right)}^{T}C_{t}\delta_{t}\left(x_{t}\right)\right){\left[\frac{\left|K_{t}\left(x_{t},x_{t}\right)\right|}{\left|A_{t}\right|}\right]}^{-1/2}{\left(2\pi \sigma_{t}^{2}\right)}^{-\gamma_{t}} \end{array}$$

with $\gamma_{t}=\frac{N_{x_{t}}-N_{\beta_{t}}}{2}$, $N_{x_{t}}$ as the number of pivot points in input vector $x_{t}$, and $N_{\beta_{t}}$ as the number of basis functions of level *t*. Apparently, the expectation of $\beta_{}$ is independent of $\sigma_{}$ and the covariance of $\beta_{}$ is independent of $\rho_{}$. We will next tend to integrating with respect to $\rho_{t-1}$. We find $\rho_{t-1}$ in $\delta_{t}\left(x_{t}\right)$ only and thus as a quadratic form again:

$$\begin{array}{cc} -\frac{1}{2\sigma_{t}^{2}}\delta_{t}{\left(x_{t}\right)}^{T}C_{t}\delta_{t}\left(x_{t}\right) & =-\frac{1}{2\sigma_{t}^{2}}\left(c_{t}-\frac{b_{t}^{2}}{a_{t}}\right)-\frac{1}{2\sigma_{t}^{2}}a_{t}{\left(\rho_{t-1}-\langle \rho_{t-1}|\alpha_{t},\sigma_{t}\rangle \right)}^{2}\\ \langle \rho_{t-1}|\alpha_{t},\sigma_{t}\rangle & =\frac{b_{t}}{a_{t}}\\ var(\rho_{t-1}|\alpha_{t},\sigma_{t}) & =\frac{\sigma_{t}^{2}}{a_{t}}\\ c_{t} & ={\left(z_{t}\left(x_{t}\right)\right)}^{T}C_{t}z_{t}\left(x_{t}\right)\\ b_{t} & ={\left(z_{t}\left(x_{t}\right)\right)}^{T}C_{t}z_{t-1}\left(x_{t}\right)\\ a_{t} & ={\left(z_{t-1}\left(x_{t}\right)\right)}^{T}C_{t}z_{t-1}\left(x_{t}\right) \end{array}$$

We can now integrate with respect to $\rho_{t-1}$. We assume a flat prior:

$$\begin{array}{cc} p\left[\alpha_{t},\sigma_{t}|D_{t}\right] & =\int p\left[\alpha_{t},\sigma_{t},\rho_{t-1}|D_{t}\right]d\rho_{t-1}\\ \; & =\exp\left(-\frac{1}{2\sigma_{t}^{2}}\left(c_{t}-\frac{b_{t}^{2}}{a_{t}}\right)\right){\left[\frac{\left|K_{t}\left(x_{t},x_{t}\right)\right|}{\left|A_{t}\right|}\right]}^{-1/2}{\left(2\pi \sigma_{t}^{2}\right)}^{-\gamma_{t}}\sqrt{\frac{2\pi \sigma_{t}^{2}}{a_{t}}} \end{array}$$

The last random variable we can treat analytically is $\sigma_{t}$. With Jeffrey’s prior $p\left[\sigma_{t}|C\right]=\frac{1}{\sigma_{t}}$, we have

$$\begin{array}{cc} p\left[\alpha_{t}|D_{t}\right] & =\int p\left[\alpha_{t},\sigma_{t}|D_{t}\right]d\sigma_{t}\\ \; & =\int \Lambda_{t}\exp\left(-\frac{\Phi_{t}}{2\sigma_{t}^{2}}\right){\left(\sigma_{t}^{2}\right)}^{-\gamma_{t}+\frac{1}{2}}\frac{d\sigma_{t}}{\sigma_{t}}\\ \Phi_{t} & =c_{t}-\frac{b_{t}^{2}}{a_{t}}\\ \Lambda_{t} & ={\left[\frac{\left|K_{t}\left(x_{t},x_{t}\right)\right|}{\left|A_{t}\right|}\right]}^{-1/2}{\left(2\pi \right)}^{-\gamma_{t}}\sqrt{\frac{2\pi }{a_{t}}} \end{array}$$

With the substitution $v=\frac{\Phi_{t}}{2\sigma_{t}^{2}}$, we find this to be a Γ-integral:

$$\begin{array}{cc} p\left[\alpha_{t}|D_{t}\right] & =\frac{\Lambda_{t}}{2}{\left(\frac{\Phi_{t}}{2}\right)}^{-\gamma_{t}+\frac{1}{2}}\underset{=\Gamma (\gamma_{t}-\frac{1}{2})}{\underset{︸}{\int e^{-v}v^{(\gamma_{t}-\frac{1}{2})-1}dv}} \end{array}$$

The moments of $\sigma_{t}$ are Γ-integrals as well, and we find

$$\begin{array}{cc} \frac{\langle \sigma_{t}^{\nu }|\alpha_{t}\rangle }{\langle \sigma_{t}^{0}|\alpha_{t}\rangle } & ={\sqrt{\Phi_{t}}}^{\nu }\frac{\Gamma (\gamma_{t}-\frac{1}{2}-\frac{\nu }{2})}{\Gamma (\gamma_{t}-\frac{1}{2})} \end{array}$$

For the above Γ-integral and the σ-moments to exist, we require $\gamma_{t}-\frac{1}{2}-\frac{\nu }{2}>0$, i.e., Equation (4d). Note that, in the case of t=1, we have no integration with respect to $\rho_{0}$ and thus find one power of $\sqrt{\sigma_{1}}$ less in the above Γ-integral, i.e., we need to substitute $\gamma_{1}-\frac{1}{2}\to \gamma_{1}$. The constraint is accordingly weakened for t=1, which is due to the absence of the single parameter $\rho_{0}$. For most choices of the covariance kernel, we cannot go further analytically.

Now, we have everything we need to marginalize $\sigma_{t}$ in the conditional expectations of $\rho_{t-1}$.

$$\begin{array}{cc} \langle \rho_{t-1}^{\nu }|\alpha_{t}\rangle & =\int \langle \rho_{t-1}^{\nu }|\alpha_{t},\sigma_{t}\rangle p\left[\alpha_{t},\sigma_{t}|D_{t}\right]d\sigma_{t} \end{array}$$

which is now relatively easy.

$$\begin{array}{cc} \langle \rho_{t-1}^{1}|\alpha_{t}\rangle & =\frac{b_{t}}{a_{t}}\\ \langle \rho_{t-1}^{2}|\alpha_{t}\rangle & =\frac{\langle \sigma_{t}^{2}|\alpha_{t}\rangle }{a_{t}}+{\left(\frac{b_{t}}{a_{t}}\right)}^{2} \end{array}$$

The expected mean function parameters are simply

$$\begin{array}{cc} \langle \beta_{t}|\alpha_{t}\rangle & =\int \langle \beta_{t}|\alpha_{t},\rho_{t-1},\sigma_{t}\rangle p\left[\alpha_{t},\rho_{t-1},\sigma_{t}|D_{t}\right]d\rho_{t-1}d\sigma_{t}\\ \; & =A_{t}t\left(z_{t}\left(x_{t}\right)-\langle \rho_{t-1}|\alpha_{t}\rangle z_{t}\left(x_{t-1}\right)\right) \end{array}$$

The variance we read from the quadratic form in $\beta_{t}$ is as follows:

$$\begin{array}{cc} var(\beta_{t}^{\left(k\right)}|\alpha_{t}) & =\langle \sigma_{t}^{2}|\alpha_{t}\rangle \cdot {\left(A_{t}\right)}_{kk} \end{array}$$

with ${\left(A_{t}\right)}_{kk}$ as the *k*th diagonal element of $A_{t}$.

### Appendix A.2. Predictive Mean

We rewrite ${\bar{\delta }}_{t}$ such that

$$\begin{array}{cc} {\bar{\delta }}_{t}\left(x\right) & =\underset{u_{t}}{\underset{︸}{K_{t}\left(x_{},x_{t}\right)K_{t}{\left(x_{t},x_{t}\right)}^{-1}\left(z_{t}\left(x_{t}\right)-\rho_{t-1}z_{t}\left(x_{t-1}\right)\right)}}+\underset{g_{t}}{\underset{︸}{\left(h_{t}\left(x\right)-K_{t}\left(x_{},x_{t}\right)K_{t}{\left(x_{t},x_{t}\right)}^{-1}h_{t}\left(x_{t}\right)\right)}}\beta_{t} \end{array}$$

The first term, $u_{t}$, assumes a rather simple form and only depends on $\rho_{t-1}$ and $\alpha_{}$. Since we found $p\left[\rho_{},\alpha_{}|D\right]=\prod_{k}p\left[\rho_{k},\alpha_{t}|D_{k}\right]$ previously, and each $\rho_{k}$ occurs linearly. We find

$$\begin{array}{cc} \langle \prod_{k}\rho_{k}|\alpha_{}\rangle & =\prod_{k}\langle \rho_{k}|\alpha_{t}\rangle \\ \Rightarrow \langle u_{t}\prod_{l=t}^{N_{t}-1}\rho_{l}|\alpha_{}\rangle & =K_{t}\left(x_{},x_{t}\right)K_{t}{\left(x_{t},x_{t}\right)}^{-1}\left(z_{t}\left(x_{t}\right)-\langle \rho_{t-1}|\alpha_{t}\rangle z_{t}\left(x_{t-1}\right)\right)\prod_{l=t}^{N_{t}-1}\langle \rho_{l}|\alpha_{t}\rangle \end{array}$$

The second term is rather trivial.

$$\begin{array}{c} \langle g_{t}\beta_{t}\prod_{l=t}^{N_{t}-1}\rho_{l}|\alpha_{t}\rangle =g_{t}\langle \beta_{t}|\alpha_{t}\rangle \prod_{l=t}^{N_{t}-1}\langle \rho_{l}|\alpha_{t}\rangle \end{array}$$

We have thus shown the predictive mean in Equation (4).

### Appendix A.3. Predictive Covariance

We easily find

$$\begin{array}{cc} \left(\sum_{t=1}^{N_{t}}\delta_{t}\left(x_{}\right)\left(x\right)\prod_{l=t}^{N_{t}-1}\rho_{l}\right){\left(\sum_{t=1}^{N_{t}}\delta_{t}\left(x_{}\right)\left(x\right)\prod_{l=t}^{N_{t}-1}\rho_{l}\right)}^{T} & =\sum_{tt^{\prime }}^{N_{t}}\delta_{t}\left(x_{}\right)\delta_{t^{\prime }}{\left(x_{}\right)}^{T}\prod_{ll^{\prime }}^{N_{t}-1}\rho_{l}\rho_{l^{\prime }} \end{array}$$

Due to the assumption of independence of the levels or each levels’ parameters, formally $\delta_{t}\left(x_{}\right)⊥z_{t-1}\left(x_{}\right)$, the cross-terms mixing different levels vanish in the expectation value and it suffices to reduce the double sum to a single sum. Further, we can factor the expectation of the product of $\rho_{l}$ and the expectation of the sum.

$$\begin{array}{cc} \langle \sum_{tt^{\prime }}^{N_{t}}\delta_{t}\left(x_{}\right)\delta_{t^{\prime }}{\left(x_{}\right)}^{T}\prod_{ll^{\prime }}^{N_{t}-1}\rho_{l}\rho_{l^{\prime }}\rangle & =\langle \sum_{t=1}^{N_{t}}\delta_{t}\left(x_{}\right)\delta_{t}{\left(x_{}\right)}^{T}\rangle \prod_{l=t}^{N_{t}-1}\langle \rho_{l}^{2}\rangle \end{array}$$

The first factor on the right-hand side can be expressed via the GP’s posterior covariance and mean:

$$\begin{array}{cc} \sum_{t=1}^{N_{t}}\langle \delta_{t}\left(x_{}\right)\delta_{t}{\left(x_{}\right)}^{T}\rangle & =\sum_{t=1}^{N_{t}}\Sigma_{t}\left(x,x\right)+\langle \delta_{t}\left(x_{}\right)\rangle {\langle \delta_{t}\left(x_{}\right)\rangle }^{T} \end{array}$$

where

$$\begin{array}{cc} \Sigma_{t}\left(x,x\right) & =\sigma_{t}^{2}\left(K_{t}\left(x_{},x_{}\right)-K_{t}\left(x_{},x_{t}\right)K_{t}{\left(x_{t},x_{t}\right)}^{-1}K_{t}\left(x_{t},x_{}\right)\right) \end{array}$$

is the GPs’ posterior covariance and the predictive mean we already know as follows:

$$\begin{array}{c} \langle \delta_{t}\left(x_{}\right)|\alpha_{t}\rangle =\langle u_{t}|\alpha_{t}\rangle +g_{t}\langle \beta_{t}|\alpha_{t}\rangle \end{array}$$

We have thus shown everything we claimed.
